# Crosslinking Liposomes/Cells Using Cholesteryl Group-Modified Tilapia Gelatin

**DOI:** 10.3390/ijms150713123

**Published:** 2014-07-23

**Authors:** Tetsushi Taguchi, Yoshiaki Endo

**Affiliations:** 1 Biomaterials Unit, Nano-Life Field, International Center for Materials Nanoarchitectonics, National Institute for Materials Science, 1-1 Namiki, Tsukuba, Ibaraki 305-0044, Japan; E-Mail: y-endo@kose.co.jp; 2 Graduate School of Pure and Applied Sciences, University of Tsukuba, 1-1-1 Tennodai, Tsukuba, Ibaraki 305-8577, Japan

**Keywords:** hydrophobically modified gelatin, cholesterol, assembly, liposome, cell

## Abstract

Cholesteryl group-modified tilapia gelatins (Chol-T-Gltns) with various Chol contents from 3 to 69 mol % per amino group of Gltn were prepared for the assembly of liposomes and cells. Liposomes were physically crosslinked by anchoring Chol groups of Chol-T-Gltns into lipid membranes. The resulting liposome gels were enzymatically degraded by addition of collagenase. Liposome gels prepared using Chol-T-Gltn with high Chol content (69Chol-T-Gltn) showed slower enzymatic degradation when compared with gels prepared using Chol-T-Gltn with low Chol content (3Chol-T-Gltn). The hepatocyte cell line HepG2 showed good assembly properties and no cytotoxic effects after addition of 69Chol-T-Gltns. In addition, the number of HepG2 cells increased with concentration of 69Chol-T-Gltns. Therefore, Chol-T-Gltn, particularly, 69Chol-T-Gltn, can be used as an assembling material for liposomes and various cell types. The resulting organization can be applied to various biomedical fields, such as drug delivery systems, tissue engineering and regenerative medicine.

## 1. Introduction

Architecture of functional nano/micro elements for three dimensional structures is a next generation of nanotechnology. In particular, three-dimensionally assembled bioelements have benefits for biomedical fields including drug delivery systems and tissue engineering. DNA [1], peptide [2] and hydroxyapatite [3] have already been assembled to obtain highly functionalized biomaterials. Liposomes have been used as a simple cell model and have been applied as drug carriers with broad clinical utility. The range of medical applications for liposome extends from chemotherapy for cancer and fungal infections to gene therapy [4,5,6]. However, administration of dispersed liposomes to specific sites in the body remains a problem. A possible solution is to immobilize liposomes inside a hydrogel network and to inject the liposome gel to the diseased area. Studies on liposomes physically crosslinked by hydrophobically modified polymers have demonstrated the possibility of entrapping liposomes within a hydrogel matrix via anchoring of the hydrophobic moieties of the polymer into the liposome bilayers. Hydrophobic moieties used include natural hydrophobic groups such as cholesterol [7,8] or synthetic hydrocarbon chains [9,10,11,12].

On the other hand, it is known that there is a significant functional difference in cells cultured on a flat layer and in a three-dimensional environment [13,14,15,16] because three-dimensional cell culture is similar to *in vivo* conditions. Hence, there have been numerous approaches for assembling cells employing various techniques and substrates, such as bioreactors [17,18,19], culture-plates [20] and microfabricated surface-controlled cell adhesion [21]. However, these require special devices and techniques, and require long periods of time for assembly. Therefore, simple and rapid methods for assembling cells are required [22,23,24].

We designed a novel biodegradable amphiphilic polymer that can assemble liposomes and cells. We employed tilapia scale-derived gelatin (T-Gltn), which has a low denaturation temperature when compared with porcine or bovine proteins as a basic material, and substituted its amino groups with cholesteryl (Chol) groups. Rheological studies were then performed by addition of Chol group-modified T-Gltn (Chol-T-Gltn) with various Chol contents in the liposome solution. Furthermore, assembly of hepatic cells using Chol-T-Gln was performed.

## 2. Results and Discussion

### 2.1. Synthesis of Cholesteryl Group-Modified Tilapia Gelatins (Chol-T-Gltns)

**Figure 1:**
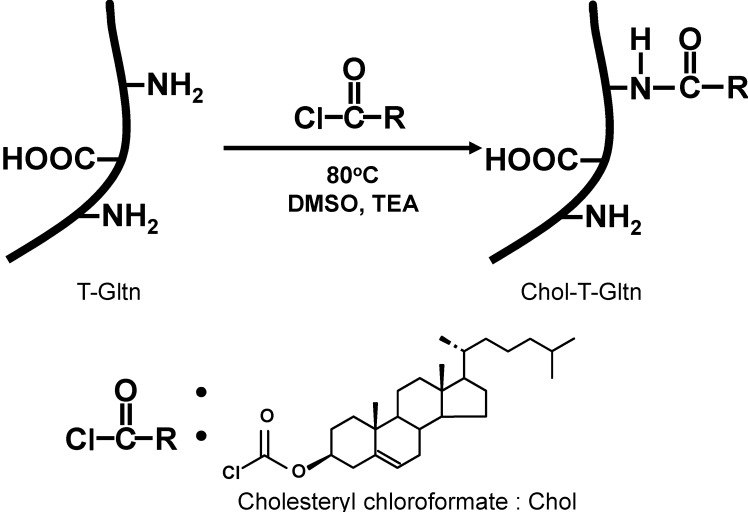
Synthesis of cholesteryl group-modified tilapia gelatin (Chol-T-Gltn).

Cholesteryl group-modified tilapia gelatins (Chol-T-Gltns) with various Chol contents from 3.2%–68.7% were successfully synthesized using a standard nucleophilic substitution reaction between the primary amine of T-Gltn and the active ester group of cholesteryl chloroformate (Figure 1, Table 1) [25]. We employed tilapia-derived gelatin for the modification with Chol groups because of its low denaturation temperature compared with porcine [26]. Most Chol-T-Gltns were quantitatively obtained, except for Chol-T-Gltn with high Chol content (69Chol-T-Gltn). The characterization of Chol-T-Gltns was performed using ^1^H-NMR, CAC, Zeta potential and spectrophotometric methods using 2,4,6-trinitrobenzensulfonic acid (TNBS). From the ^1^H-NMR measurement of Chol-T-Gltn, the distinguishing signal of the Chol group was observed at 0.65 ppm (^1^H, ^18^C) after the reaction, indicating that the Chol group was introduced into the T-Gltn molecule. In addition, CAC in Chol-T-Gltns decreased with increasing Chol contents. From the measurement of Zeta potential of Chol-T-Gltns, negative charges increased with increasing Chol contents, indicating that Chol-T-Gltns became negatively charged polymers due to the substitution reaction of primary amino groups with Chol groups. The results from ^1^H-NMR, CAC, Zeta potential and amino group determination using TNBS method suggested that the amino groups in T-Gltn were partially converted to Chol groups.

**Table 1:** Characteristics of Chol group-modified tilapia gelatin.

Abbreviation	Chol Content (mol %)	CAC (mg/L)	Zeta Potential (mV)	Yield (%)
00Chol-T-Gltn	–	36.5	6.02	–
03Chol-T-Gltn	3.2	24.9	−12.10	91.6
11Chol-T-Gltn	11.3	6.63	−2.81	88.6
19Chol-T-Gltn	19.2	5.64	−11.43	83.9
44Chol-T-Gltn	44.4	4.51	−16.30	85.8
69Chol-T-Gltn	68.7	3.42	−11.18	60.9

### 2.2. Crosslinking of Liposome with Chol-T-Gltns

**Figure 2:**
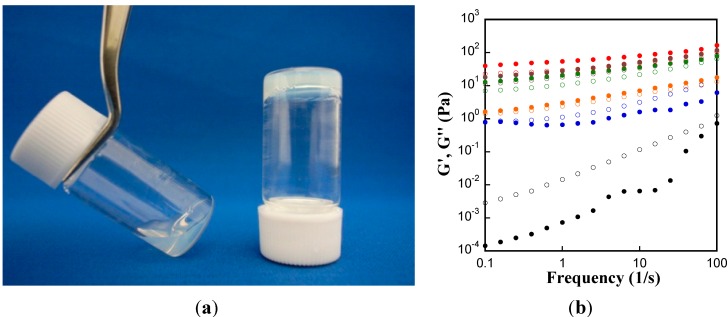
Assembly of liposome with Chol-T-Gltns. (**a**) Photograph of Dimethyldioctadecylammonium bromide (DODAB) liposome solution (20 mM) before (**left**) and after (**right**) addition of Chol-T-Gltn with high Chol content (69Chol-T-Gltn) (20 *w*/*v* %); and (**b**) Storage modulus G' (closed symbol) and loss modulus G'' (open symbol) of mixture solution of liposome with Chol-T-Gltns as a function of frequency at 37 °C. Chol contents of T-Gltns were 0 (●,○), 3 (●,○), 11 (●,○), 19 (●,○), 44 (●,○), and 69 (●,○) mol %, respectively.

In order to confirm liposome assembly, liposome solutions were mixed with Chol-T-Gltns. In this study, Dimethyldioctadecylammonium bromide (DODAB) was selected for the preparation of liposome solution. DODAB is the synthetic double-chained cationic lipid that behaves similarly to biomembrane lipids in an aqueous environment. As can be seen from Figure 2a, DODAB liposome solution was a bluish, translucent dispersion, characteristic of small unilamellar vesicles (Figure 2a, left). After addition of 69Chol-T-Gltn to DODAB liposome solution, it transformed into an elastic gel-like solid that could hold its own weight on gravity (Figure 2a, right). Cholesterol is a naturally occurring lipid and can be metabolized in the body. Due to its hydrophobicity and biocompatibility, Chol groups were successfully introduced into both natural biopolymers such as pullulan [27], chitosan [28] and synthetic polymers such as polyethylenimine [29] for various applications, such as protein or gene delivery, surface modifiers for liposomes [28] and nanoparticles [30]. In this liposome gel, Chol groups of 69Chol-T-Gltn may anchor into the bilayers of liposomes via hydrophobic interactions thus assembly of liposome. Similar research has been done by Meier, W. *et al.* [7]. They crosslinked DODAB using cholesterol group-modified poly(ethylene glycol) and confirmed that DODAB liposomes still retained their shape even after crosslinking. Therefore, DODAB liposomes still have a spherical shape after crosslinking with 69Chol-T-Gltn.

In order to confirm gel formation, dynamic moduli (storage modulus G' and loss modulus G'') as a function of the applied frequency were assessed in frequency sweep experiments using mixture solutions composed of liposome solution and Chol-T-Gltns with various Chol contents at 37 °C. The final concentrations of liposome and Chol-T-Gltns in mixture solutions were fixed at 20 mM and 20 *w*/*v* %, respectively. As shown in Figure 2b, G'' exceeded G' over the entire frequency spectra when 0Chol-T-Gltn and Chol-T-Gltn with low Chol content (3Chol-T-Gltn) were used, indicating that the mixture was in a sol state. On the other hand, with addition of Chol-T-Gltns whose Chol contents were more than 11 mol %, G' predominated in higher frequency spectra than G'', revealing that liposome gel could be formed by addition of Chol-T-Gltns.

### 2.3. Effect of β-Cyclodextrin (β-CD) Addition on Liposome Crosslinking

**Figure 3:**
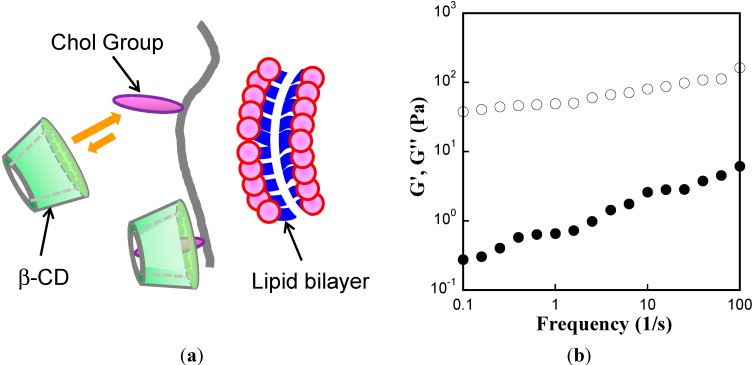
Prevention experiment of liposome gel formation by addition of β-cyclodextrin (β-CD). (**a**) A schematic illustration of capping Chol groups in Chol-T-Gltn by β-CD; and (**b**) Storage modulus G' (●) and loss modulus G'' (○) of DODAB liposome gel as a function of frequency at 37 °C. Concentrations of DODAB liposome, 69Chol-T-Gltn and β-CD were fixed at 20 mM, 20 *w*/*v* % and 23.5 mM, respectively.

We also confirmed that the Chol groups of Chol-T-Gltn were anchored into the lipid membrane of DODAB. For this purpose, a sugar-based supramolecule, β-cyclodextrin (β-CD), was employed. β-CD is a barrel-shaped supramolecule that has a hydrophobic interior pocket. This hydrophobic pocket can encapsulate Chol groups in amphiphilic polymers (Figure 3a) in a water environment. Using 69Chol-T-Gltn, we attempted to confirm liposome gel formation in the presence of β-CD. The concentration of β-CD was fixed at 23.5 mM. Figure 3b shows the G' and G'' values of DODAB liposome solution as a function of frequency at 37 °C. G'' exceeded G' throughout the frequency spectra, indicating that liposome gel formation by 69Chol-T-Gltn was fully inhibited by the addition of β-CD. In other words, the Chol groups in Chol-T-Gltns anchor into the lipid membranes of DODAB liposomes to form liposome gel.

### 2.4. Enzymatic Degradation of Liposome Gel

**Figure 4:**
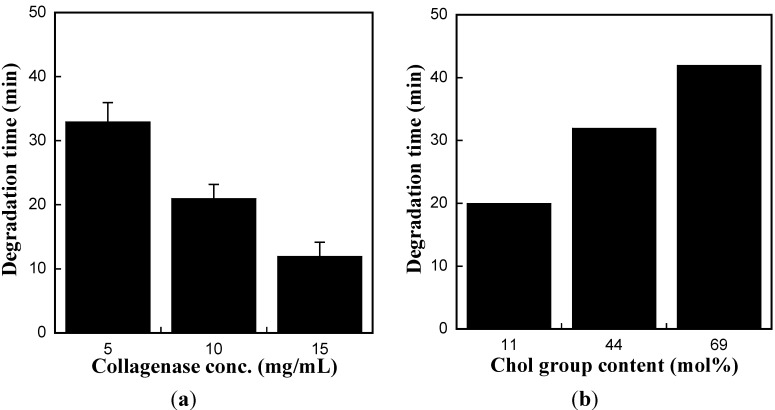
Enzymatic degradation study of Chol-T-Gltn crosslinked DODAB liposome gel. (**a**) Effects of collagenase concentration on degradation time. Data are means ± SD of three samples. 40 *w*/*v* % 44Chol-T-Gltn solution was mixed with 40 mM DODAB liposome solution. Mixing ratio was 1/1 (*v*/*v*); and (**b**) Effects of Chol group content on degradation time. Collagenase concentration was fixed at 5 mg/mL.

Chol-T-Gltns have a gelatin-based backbone; therefore, liposome gels prepared with Chol-T-Gltns will have the potential for enzymatic degradability. To confirm this hypothesis, degradation behavior was observed using rheometer. We defined degradation time of Chol-T-Gltn-crosslinked liposome when G' value is equal to G''. Figure 4a shows the effects of collagenase concentration on degradation time. In this experiment, 44Chol-T-Gltn-crosslinked liposome was employed. Degradation of liposome gels increased with collagenase concentration. This means that the decrease in molecular weight of 44Chol-T-Gltn occurred rapidly at higher collagenase concentrations. We also performed a collagenase degradation study of liposome gels using various Chol-T-Gltns with different Chol contents. As can be seen from Figure 4b, degradation time increased with Chol content of Chol-T-Gltns at the same collagenase concentrations. This may be due to the fact that physical crosslinking densities increased when Chol-T-Gltn with high Chol contents was used for the preparation of liposome gels.

### 2.5. Crosslinking of HepG2 Cells with Chol-T-Gltns

**Figure 5:**
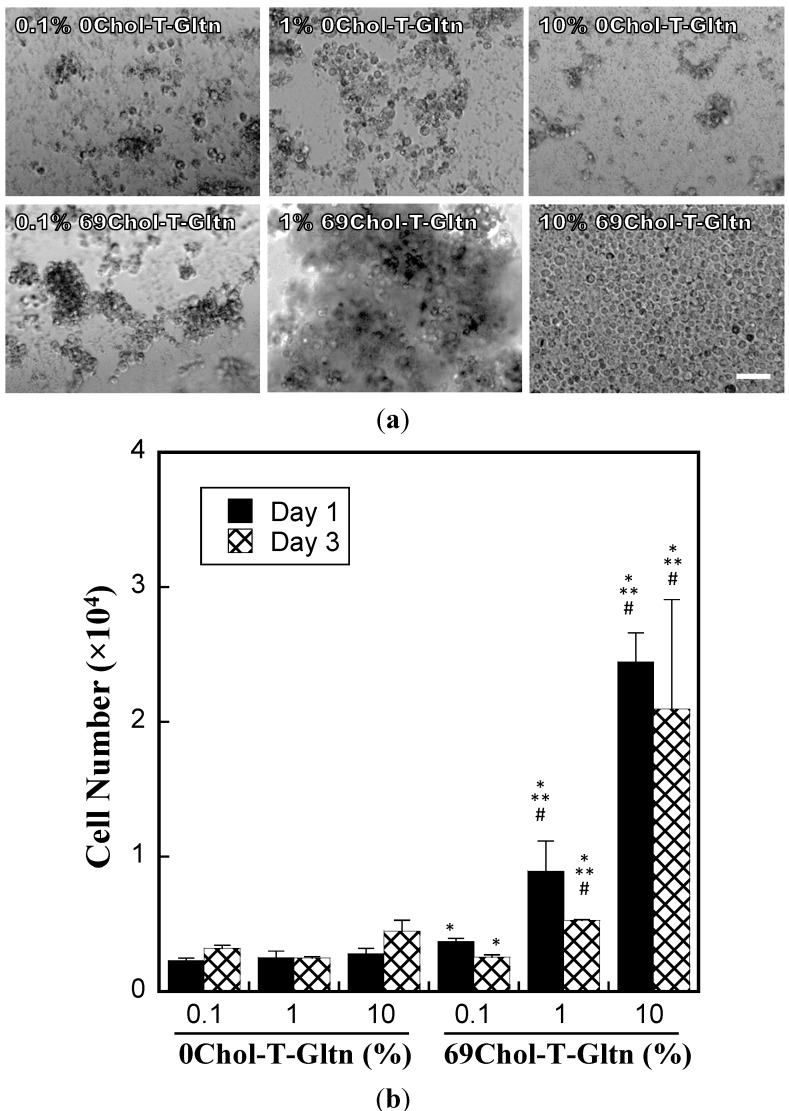
Assembly of HepG2 cells by addition of 0Chol-T-Gltn or Chol-T-Gltn with high Chol content (69Chol-T-Gltn). (**a**) Phase contrast micrograph of HepG2 cells after addition of 0Chol-T-Gltn or 69Chol-T-Gltn with various concentrations.Scale bar = 50 μm; and (**b**) Number of HepG2 cells after culture for 1 or 3 days at different 0Chol-T-Gltn or 69Chol-T-Gltn concentrations. Data are means ± SD of three samples. * *p* < 0.05 *vs.* same concentration of 0Chol-T-Gltn; ** *p* < 0.05 *vs.* 0.1% 69Chol-T-Gltn (**Day 1**); # *p* < 0.05 *vs.* 0.1% 69Chol-T-Gltn (**Day 3**).

We also attempted to form cell assemblies using Chol-T-Gltn. HepG2 cells were employed as a typical cell line. Cells were suspended in medium without serum and 0Chol-T-Gltn or 69Chol-T-Gltn solution was added. The mixture was then stirred with a micromixer, followed by placing the cell aggregates into a plate for culture. Figure 5a shows the phase-contrast micrographs of HepG2 cells in the presence of different concentrations of 0Chol-T-Gltn or 69Chol-T-Gltn after culture for 1 day. In the case of 0Chol-T-Gltn, cells were distributed over the surface of the culture plate, even at high concentrations of 0Chol-T-Gltn. Cell assembly was confirmed when 69Chol-T-Gltn was added to the cells, and firm cell assembly was observed after culture for 1 day when the concentration of 69Chol-T-Gltn was 10%. In our previous study, HepG2 cell assembly was confirmed when cells were cultured with di-oleoyl group-terminated poly (ethylene) glycol (Ole-PEG-Ole) [24]. This Ole-PEG-Ole can also play a similar role as Chol-T-Gltns, that is, hydrophobic groups such as Ole or Chol groups anchor to the cell membrane, resulting in cell assembly. However, it took 7 days to assemble cells when Ole-PEG-Ole was added to HepG2 cells. These differences were due to the presence of cell adhesion sequences in the 69Chol-T-Gltn molecule. The backbone of Chol-T-Gltn was gelatin, which has the cell adhesion sequence RGD (Arg-Gly-Asp). After Chol groups of Chol-T-Gltn anchor to the phospholipid cell membrane, integrin in HepG2 cells recognizes the RGD sequence of Chol-T-Gltn, resulting in the promotion of cell assembly. On the other hand, Ole-PEG-Ole can also anchor to the phospholipid cell membrane and physically crosslink among cells, however, cells cannot recognize PEG because of its high hydrophilicity. Due to these differences, aggregated HepG2 cells could be obtained rapidly after the addition of 69Chol-T-Gltn. Furthermore, we have already reported the behavior of FITC-labeled cholesteryl groups modified Gltn after addition to the cells [25]. FITC-labeled non-modified Gltn was used as a control. Nuclei of cells were also stained with DAPI. From those experiments, it was clear that cholesteryl groups-modified Gltn was highly integrated on the surface of the cells compared to non-modified Gltn. These previous data supported that 0Chol-T-Gltn cannot effectively adhere onto the surface of the cells compared to 69Chol-T-Gltn. Therefore, cell proliferation was stimulated by the addition of 69Chol-T-Gltn compared to 0Chol-T-Gltn.

After culture for 1 or 3 days, we determined the number of HepG2 cells cultured with 0Chol-T-Gltn or 69Chol-T-Gltn using Cell Counting Kit-8. As shown in Figure 5b, the number of HepG2 cells was increased by the addition of 69Chol-T-Gltn at all concentrations when compared with 0Chol-T-Gltn (*p* < 0.05). After culture for 1 day, the number of HepG2 cells increased with concentration of 69Chol-T-Gltn. A similar phenomenon was observed with HepG2 cells cultured in the presence of 69Chol-T-Gltn for 3 days. The 69Chol-T-Gltn used in this study is an amphiphilic polymer with a critical micelle concentration of 3.42 mg/L (0.000342%) at 37 °C, as shown in Table 1. Therefore, 69Chol-T-Gltn formed micelles in cell culture medium. Under these conditions, 69Chol-T-Gltn was expected to exhibit toxicity against HepG2 cells; however, no significant decreases in cell number were observed during the culture periods. In contrast, 69Chol-T-Gltn had a positive effect on the proliferation of HepG2 cells; the number of cells increased with concentration of 69Chol-T-Gltn. This may be due to the cytocompatibility of Gltn chain. The RGD sequence of 69Chol-T-Gltn also worked as a scaffold for cell adhesion and proliferation. We previously added Ole-PEG-Ole to HepG2 cells with different concentrations [24]. However, the number of HepG2 cells decreased with increasing concentration of Ole-PEG-Ole, even though cell function was promoted. These results indicated that hydrophobic groups such as Ole or Chol groups are effective for cell assembly and that the cell adhesive properties of main chains such as Gltn are required for cytocompatibility after cell assembly.

## 3. Experimental Section

### 3.1. Materials

Gelatin from tilapia scales (T-Gltn, type B, *M*_W_ = 70,000) was kindly donated by Nitta Gelatin Inc. (Osaka, Japan). Triethylamine (TEA), sodium dodecyl sulfate (SDS), dehydrated dimethylsulfoxide (DMSO), ethanol, ethyl acetate, 2,4,6-trinitrobenzensulfonic acid, hydrochloric acid, tartaric acid and β-cyclodextrin (β-CD) were purchased from Wako Pure Chemical Industries, Ltd. (Osaka, Japan). Cholesteryl chloroformate was purchased from Sigma Chemical Co. (St. Louis, MO, USA). Dimethyldioctadecylammonium bromide (DODAB, >98%) was purchased from Sigma–Aldrich (St. Louis, MO, USA). Collagenase (130 U/mg, solid) was purchased from Nacalai Tesque, Inc. (Kyoto, Japan). All chemicals were used without further purification.

### 3.2. Synthesis and Characterization of Chol-T-Gltn

Modification of T-Gltn with Chol groups was carried out using a reaction between the active ester of cholesteryl chloroformate with the primary amine of T-Gltn . Briefly, a mixture of T-Gltn (40 g) and TEA (5 mL) in DMSO (495 mL) was stirred at 80 °C. Powdered cholesteryl chloroformate was then added to the T-Gltn solution. The mixture continued to be stirred under an N_2_ atmosphere at 80 °C overnight. The resulting Chol-T-Gltn was precipitated by adding three volumes of cold ethanol. Precipitates were subsequently washed three times with 1.5 L of ethyl acetate in order to remove unreacted cholesteryl chloroformate and DMSO. The resulting solid was dried at 25 °C to leave a white cake.

Introduction of the Chol group into T-Gltn was confirmed by ^1^H-NMR (AL300; JEOL, Tokyo, Japan). Critical aggregation concentration (CMC) of Chol-T-Gltn was also measured by fluorescence analysis using a spectrofluorometer (FP-6500; JASCO, Tokyo, Japan) and z-potential was determined by electrophoretic light scattering (ELS) (ELS-6000; Otsuka Electronics, Osaka, Japan). The degree of substitution of amino groups with Chol groups was determined by a spectrophotometric method using TNBS. Briefly, 50 μL of 0.1% Chol-T-Gltn, 25 μL of 0.1% SDS, 50 μL of 0.1% TEA and 50 μL of 0.1% TNBS were placed in a 48-well plate, followed by incubation at 37 °C for 2 h. After adding 25 μL of 2 N HCl, each sample was measured spectrophotometrically at 340 nm using a microplate reader (GENios A-5082; Tecan, Tokyo, Japan).

### 3.3. Preparation of Liposome Solution

DODAB liposome solution was prepared using ultrasonication in a water bath and subsequent extrusion above the main transition temperature (Tm = 45 °C) through 100 nm PC membrane according to the instructions of Avanti Polar Lipid, Inc (Alabaster, AL, USA). The liposome solution was then left to cool down to room temperature before use.

### 3.4. Preparation and Characterization of Liposome Gels

We then mixed 40 *w*/*v* % Chol-T-Gltns/HEPES buffer solution and liposome/HEPES buffer solution (40 mM) in a vortex mixer, followed by gentle mixing at 37 °C for 2 h. Dynamic rheological experiments were performed using an MCR 301 rheometer (Anton Paar GmbH, Graz, Austria) equipped with parallel-plate geometry (diameter: 25 mm) and a solvent trap. Frequency sweep experiments were carried out in the linear viscoelastic range, as determined by strain sweep experiments. Frequencies varied from 0.1 to 100 rad/s. Strain sweep experiments were performed with a fixed frequency of 10 rad/s.

### 3.5. Enzymatic Degradation Study of Liposome Gels

Enzymatic degradation study of liposome gels was performed using the following procedure. Forty *w*/*v* % of 11, 44, 69Chol-T-Gltn solution was prepared with 0.1 M Tris–HCl buffer solution (pH 7.4, 2.5 mM calcium chloride). Liposome solution (40 mM) was also prepared in 0.1 M Tris–HCl buffer solution containing 5, 10, 15 μg/mL (5, 10, 15 units) collagenase at 37 °C. Liposome and Chol-T-Gltn solutions were then mixed together at volume ratio 1:1. Degradation time (G' = G'') of liposome gels was then determined using a rheometer.

### 3.6. Crosslinking of HepG2 Cell with Chol-T-Gltn

The human hepatocellular carcinoma cell line HepG2 was used for cell assembly experiments. HepG2 cells were grown in Dulbecco modified Eagle medium (DMEM; plus 4500 mg·glucose·L^−1^; Sigma–Aldrich, St. Louis, MO, USA) containing 2% penicillin-streptomycin (Invitrogen, Carlsbad, CA, USA) and 10% fetal bovine serum (FBS) (JRH Biosciences, Lenexa, KS, USA). Experimental procedures were as follows: 100 μL of HepG2 cells (1 × 10^6^ cells·mL^−1^) was suspended in medium without serum in a 1.5 mL microtube. A volume of 100 μL of 0Chol-T-Gltn or 69Chol-T-Gltn (0.1–10 *w*/*v* %; sterilized under ultraviolet light for 15 min) dissolved in phosphate buffer was added to microtubes, and the mixture was stirred with a micromixer for 2 min followed by placement of cell aggregates into the wells of a 48-well culture plate. The mixture was cultured in a humidified incubator (ESPEC Corp., Osaka, Japan) at 37 °C under a 5% CO_2_ atmosphere. Half of the culture medium was replaced every 2 days with DMEM containing 10% FBS. The number of cells cultured with 0Chol-T-Gltn or 69Chol-T-Gltn was determined using Cell Counting Kit-8 (Dojindo, Kumamoto, Japan) in accordance with the manufacturer’s instructions.

### 3.7. Statistical Analysis

Results are expressed as means ± standard deviation (SD). Data were analyzed by Student’s *t*-test using the statistical function of Microsoft Excel. All *p* values were compared to a value of 0.05 to determine significance.

## 4. Conclusions

For the assembly of liposomes and cells, Chol groups-modified tilapia gelatins (Chol-T-Gltns) with various Chol contents were prepared. Liposomes were physically crosslinked by anchoring Chol groups to lipid membranes. The resulting liposome gels showed enzymatic degradation properties, and degradation rate could be controlled by changing the Chol contents of Chol-T-Gltns. 69Chol-T-Gltns promoted assembly of hepatic cells and showed excellent cytocompatibility. 69Chol-T-Gltns can therefore be used as an assembling material for liposomes or various cell types. The resulting organization can then be applied to various biomedical fields, such as drug delivery systems, tissue engineering and regenerative medicine.
